# Prioritising primary care patients with unexpected weight loss for cancer investigation: diagnostic accuracy study (update)

**DOI:** 10.1136/bmj-2024-080199

**Published:** 2024-10-16

**Authors:** Brian D Nicholson, Pradeep Virdee, Paul Aveyard, Sarah J Price, F D Richard Hobbs, Constantinos Koshiaris, Willie Hamilton

**Affiliations:** 1 Nuffield Department of Primary Care Health Sciences, University of Oxford, Oxford, UK; 2 Medical School, University of Exeter, Exeter, UK

## Abstract

**Objective:**

To quantify the predictive value of unexpected weight loss for cancer according to patient’s age, sex, smoking status, and concurrent clinical features (symptoms, signs, and abnormal blood test results).

**Design:**

Diagnostic accuracy study (update).

**Setting:**

Data from Clinical Practice Research Datalink electronic health records linked to the National Cancer Registration and Analysis Service in primary care, England.

**Participants:**

326 240 adults (≥18 years) with a code for unexpected weight loss from 1 January 2000 to 31 December 2019.

**Main outcome measures:**

Cancer diagnosis in the six months after the earliest weight loss code (index date). Codes for additional clinical features were identified in the three months before to one month after the index date. Diagnostic accuracy measures included positive and negative likelihood ratios, positive predictive values, and diagnostic odds ratios.

**Results:**

Of 326 240 adults with unexpected weight loss, 184 270 (56.5%) were women, 176 508 (54.1%) were aged ≥60 years, and 176 053 (54.0%) were ever smokers. 15 624 (4.8%) had a diagnosis of cancer within six months of the index date, of whom 15 051 (96.3%) were aged ≥50 years. The positive predictive value for cancer was above the 3% threshold recommended by the National Institute for Health and Care Excellence for urgent investigation in men aged ≥50 years and women aged ≥60 years. 17 additional clinical features were associated with cancer in younger men with unexpected weight loss, and eight in women. Positive likelihood ratios in men ranged from 1.43 (95% confidence interval 1.30 to 1.58) for fatigue to 21.00 (8.59 to 51.37) for rectal mass, and in women from 1.28 (1.16 to 1.41) for back pain to 19.46 (12.69 to 29.85) for pelvic mass. Abnormal blood test results associated with cancer included low albumin (positive likelihood ratio 3.24, 3.13 to 3.35) and raised platelets (3.48, 3.35 to 3.62), total white cell count (3.01, 2.89 to 3.14), and C reactive protein (3.13, 3.05 to 3.20). However, no normal blood test result in isolation ruled out cancer. Clinical features co-occurring with unexpected weight loss were associated with multiple cancer sites.

**Conclusion:**

The risk of cancer in younger adults with unexpected weight loss presenting to primary care is <3% and does not merit investigation under current UK guidelines. However, in men aged ≥50 years, women aged ≥60 years, and younger patients with concurrent clinical features, the risk of cancer warrants referral for invasive investigation. Clinical features typically associated with specific cancer sites are markers of several cancer types when they occur with unexpected weight loss.

**Readers’ note:**

This article is an updated version of a previously published BMJ paper that has since been retracted.

## Introduction

Unexpected weight loss is associated with both early and late stage cancers in adults attending primary care.^1^ ^2^ ^3^ The likelihood of a cancer diagnosis in people with unexpected weight loss is increased in the three to six months after the first record of the weight change compared with people without unexpected weight loss: men with unexpected weight loss are three times as likely as men without unexpected weight loss to have a diagnosis of cancer within three months and are twice as likely to receive a diagnosis within six months; women with unexpected weight loss are twice as likely to have a diagnosis of cancer within three months.^1^ The greatest risks are from lymphoma, cancer of unknown primary, or cancers of the pancreas, gastro-oesophageal tract, lung, bowel, or renal tract.^1^ ^4^ A cancer diagnosis is less likely than in people without recorded unexpected weight loss after the initial three to six month period.^1^

Unexpected weight loss can also be caused by a wide range of benign and serious conditions associated with various bodily systems, lifestyle choices, and socioeconomic factors.^2^ Differential diagnoses include advanced heart failure, chronic obstructive pulmonary disease, renal disease, pancreatic insufficiency, malabsorption, and endocrine disease, with up to 25% of patients without a diagnosis to explain their weight loss after extended follow-up.^2^ ^5^ The non-specific nature of unexpected weight loss creates the clinical problem of who should be investigated further for cancer—and possibly using invasive methods—and who could be spared investigation. Several clinical reviews have proposed plausible approaches assessing the risk of cancer, but evidence generally has been from studies of older people admitted to hospital for investigation.^2^ Such research does not directly help general practitioners to plan investigations in primary care because of spectrum bias.^6^ Given the absence of appropriate clinical guidelines, or standardised practice, doctors have been reported to take diverse action, from doing nothing to ordering “extensive blind investigations” to avoid missing underlying cancer.^7^ ^8^

Most research on the predictive value of cancer related unexpected weight loss in primary care has included patients on the basis of their final cancer diagnosis rather than on weight loss.^4^ The evidence base informed the National Institute for Health and Care Excellence guidance on suspected cancer, which recommended further investigations for patients with a positive predictive value (PPV) for cancer that exceeded 3%.^9^ Studies have investigated unexpected weight loss together with other symptoms and signs occurring over a one to two year period preceding the cancer diagnosis without acknowledging that the predictive value of individual symptoms will vary during different periods.^4^ ^10^ ^11^ In this context, predictive values could have been reported for pairs of clinical features that occurred months or years apart, potentially with different causes unrelated to the eventual cancer diagnosis.

Although simple blood tests are often used to investigate non-specific symptoms in primary care patients,^12^ ^13^ ^14^ the role of such tests in selecting those with unexpected weight loss for further investigation of cancer is poorly understood. Abnormal test results might facilitate patient triage,^15^ ^16^ be poor predictors of cancer,^17^ ^18^ or be predictive across several cancer sites.^19^ Triage testing in primary care is important to avoid unnecessary urgent referrals of patients for invasive investigation.

We conducted a diagnostic accuracy study using routinely collected electronic health records in primary care to establish the predictive value of unexpected weight loss for cancer, given the patient’s age, sex, smoking status, concurrent symptoms or signs, and blood test results. To identify malignancies that might be prioritised for further investigation after referral, we considered the predictive value for cancer overall and by cancer site.

This article is an updated version of a previously published BMJ paper, which has since been retracted (doi:10.1136/bmj.m2651). The authors identified a selection bias in the previous paper, which meant that some patients were excluded from the study because their healthcare records contained a code that was not associated with unexpected weight loss. Sometime later, however, some of these patients might have had a code included in their healthcare record that was associated with unexpected weight loss and cancer. This resulted in the previous study underestimating the prevalence of cancer in patients with unexpected weight loss attending primary care, which modified the study’s key results and messages.

## Methods

### Study design and population

We performed a retrospective diagnostic accuracy study using electronic health records from Clinical Practice Research Datalink (CPRD), a representative database of anonymised primary care records covering 13% of the UK population,^20^ linked to the National Cancer Registrations and Analysis Service (NCRAS) cancer register. The “Performance of diagnostic strategies” section of the published protocol pertains to this analysis.^8^ We followed the RECORD (reporting of studies conducted using observational routinely collected data) reporting guidelines.^21^ Study entry was from 1 January 2000 to 31 December 2019 to allow two years or more to accommodate the time it takes for NCRAS to release validated data.

Patients were included if they were aged ≥18 years; registered with a general practice contributing data to CPRD and eligible for linkage to NCRAS, Hospital Episode Statistics, and Office for National Statistics data; and had at least one code for unexpected weight loss and at least 12 months of data before the first recorded unexpected weight loss code (the index date). These unexpected weight loss codes equated to a mean weight loss of ≥5% within a six month period in our previous internal validation study of weight related coding in CPRD.^22^ Unexpected weight loss could be coded according to a range of clinical scenarios, including unexpected weight loss reported as the patient’s presenting condition with or without objective evidence, after targeted history taking by the clinician, and after weight measurement as part of the clinical examination or as part of a routine health check or chronic disease review.

We excluded patients if they had a prescription of weight reducing treatment (orlistat) or a code for bariatric surgery in the previous six months, or if they had a cancer diagnosis before the index date.

### Cancer (reference standard)

To identify cancers, we updated an existing library of codes to include all high level ICD-O (international classification of diseases for oncology) categories.^1^ We excluded cancers classified as non-melanoma skin, in situ, benign, ill defined, or uncertain. Furthermore, we grouped cancer sites that would usually be investigated using the same test or by the same specialty—for example, renal, ureteric, and bladder as renal tract cancers, and liver, gallbladder, and biliary tree as hepatobiliary cancers. All cancers diagnosed in the six months after the index date were identified in CPRD and linked NCRAS data. We used the first site specific cancer code after the index date to define cancer site. Cancer of unknown primary was defined if a code identifying a secondary cancer (such as lymph node metastasis or cerebral metastasis) was found but there was no code for a primary cancer.

### Sociodemographic and clinical features

Sociodemographic details coded on or before the index date were extracted from CPRD records. We identified codes related to signs and symptoms and blood test results in the three months before the index date to one month after. A long list of symptoms and signs shown to have an independent association with undiagnosed cancer were selected either through their inclusion in the NICE NG12 guidance for suspected cancer or based on studies published after the NICE guidance—that is, central nervous system malignancies and head and neck cancers (see supplementary appendix 1). For blood tests, we identified those most commonly requested within the four month period and dropped outliers and dichotomised continuous test results as abnormal or normal using standard laboratory ranges (see supplementary appendix 2).

### Statistical analysis

Box 1 shows the definitions of true and false positive and negative test results for clinical features. For combinations of unexpected weight loss and age group, sex, smoking status, clinical features, and abnormal blood test results, we estimated diagnostic accuracy statistics for the cancer outcome using 2×2 tables with the DIAGT Stata module: positive likelihood ratios, negative likelihood ratios, PPVs, and diagnostic odds ratios along with 95% confidence intervals (CIs). A rule of thumb is that a test with a positive likelihood ratio ≥5 is reliable for ruling in disease, and a test with a negative likelihood ratio ≤0.2 is reliable for ruling out disease.^23^ ^24^ The analysis was conducted for cancer overall and by cancer site. To select symptoms and signs, we used multivariable backwards stepwise logistic regression starting with all symptoms, signs, and sociodemographic factors as independent covariates, using a value of P≤0.01 for retention (see supplementary appendix 1). We elected to use an indicator variable over multiple imputation to replace missing data on lifestyle factors, as the main purpose of including the covariates was to reduce confounding in variable selection rather than to identify the association between the lifestyle covariate with missing data and cancer.

Box 1 **Classification of true and false positive and negative test results**

**True positive result**

Presence of a clinical feature recorded in CPRD in the three months before to one month after the index date of unexpected weight loss in patients with a cancer diagnosis (recorded in CPRD or NCRAS cancer registry) in the six months after the index date

**False positive result**

Presence of a clinical feature recorded in CPRD in the three months before to one month after the index date of unexpected weight loss in patients with no cancer diagnosis (recorded in CPRD or NCRAS cancer registry) in the six months after the index date

**False negative result**

Absence of a clinical feature recorded in CPRD in the three months before to one month after the index date of unexpected weight loss in patients with a cancer diagnosis (recorded in CPRD or NCRAS cancer registry) in the six months after the index date

**True negative result**

Absence of a clinical feature recorded in CPRD in the three months before to one month after the index date of unexpected weight loss in patients with no cancer diagnosis (recorded in CPRD or NCRAS cancer registry) in the six months after the index date

CPRD=Clinical Practice Research Datalink; NCRAS=National Cancer Registrations and Analysis Service

In discrete analyses we also calculated diagnostic accuracy statistics for each of the 11 most frequently recorded blood tests, and we included only patients with a test result for each. When tests were components of another test, we chose the quantum—for example, using total white cell count rather than white cell subtypes.

### Sensitivity analysis

We repeated the selection process for clinical features using an interval of three months before the index date to the first date of either three months after or the cancer diagnosis to explore whether broadening the window for inclusion of clinical features changed our findings.

### Patient and public involvement

Patients and members of the public were involved in an advisory capacity in the application for funding to support this research. An advisory panel of patients and members of the public provided comments on a related article that informed the current analysis. Patients were not directly involved in the conduct or analysis of the study. 

## Results

**Fig 1:**
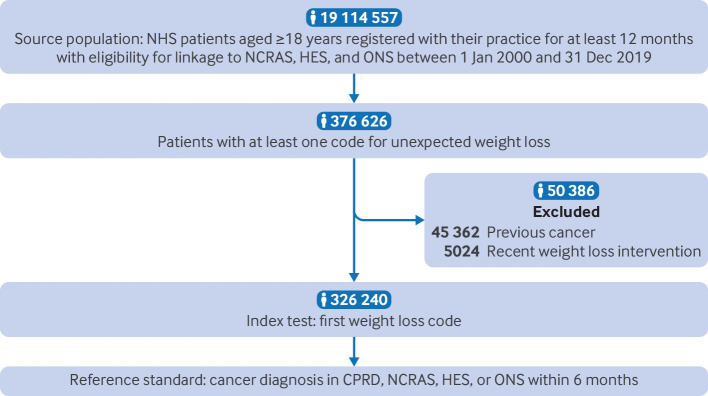
Flow of participants through study. CPRD=Clinical Practice Research Datalink; HES=Hospital Episode Statistics; NCRAS=National Cancer Registrations and Analysis Service; ONS=Office for National Statistics

Figure 1 shows the flow of participants through the study. Of 326 240 patients with a code for unexpected weight loss, 184 270 (56.5%) were women, 176 508 (54.1%) were aged ≥60 years, 142 654 (43.7%) had a body mass index (BMI) within normal range, and 176 053 (54.0%) were ever smokers (table 1). The most commonly recorded features alongside unexpected weight loss were cough (6.7%), abdominal pain (5.4%), back pain (5.3%), shortness of breath (5.3%), and chest infection (4.7%) (see supplementary appendix 3). The most frequently recorded tests were full blood count (predominantly for haemoglobin (68.4%), platelets (67.7%), and total white cell count (67.8%)), and liver function tests—namely, bilirubin (63.8%), albumin (61.2%), alkaline phosphatase (64.0%), and alanine transaminase/aspartate transaminase (63.7%) (see supplementary appendix 2).

**Table 1:** Baseline characteristics of study population

Characteristics	No (%)		
	Women (n=184 270)	Men (n=141 970)	Overall (n=326 240)
**Age group (years)**			
18-39	42 600 (23.1)	25 383 (17.9)	67 983 (20.8)
40-49	20 699 (11.2)	17 657 (12.4)	38 356 (11.8)
50-59	21 483 (11.7)	21 910 (15.4)	43 393 (13.3)
60-69	23 687 (12.9)	24 169 (17.0)	47 856 (14.7)
70-79	33 426 (18.1)	28 513 (20.1)	61 939 (19.0)
≥80	42 375 (23.0)	24 338 (17.1)	66 713 (20.4)
**Smoking status**			
Current smoker	56 983 (30.9)	46 152 (32.5)	103 135 (31.6)
Former smoker	34 777 (18.9)	38 141 (26.9)	72 918 (22.4)
Non-smoker	32 649 (17.7)	18 047 (12.7)	50 696 (15.5)
Missing	59 861 (32.5)	39 630 (27.9)	99 491 (30.5)
**Alcohol status**			
Current drinker	75 490 (41.0)	75 497 (53.2)	150 987 (46.3)
Former drinker	170 (0.1)	185 (0.1)	355 (0.1)
Non-drinker	54 882 (29.8)	28 414 (20.0)	83 296 (25.5)
Missing	53 728 (29.2)	37 874 (26.7)	91 602 (28.1)
**Body mass index**			
Underweight	14 238 (7.7)	5591 (3.9)	19 829 (6.1)
Normal	84 249 (45.7)	58 405 (41.1)	142 654 (43.7)
Overweight	35 884 (19.5)	38 025 (26.8)	73 909 (22.7)
Obese	24 589 (13.3)	17 359 (12.2)	41 948 (12.9)
Missing	25 310 (13.7)	22 590 (15.9)	47 900 (14.7)
**Cancer diagnosis**			
Yes	6161 (3.3)	9463 (6.7)	15 624 (4.8)
No	178 109 (96.7)	132 507 (93.3)	310 616 (95.2)

### Age, sex, and smoking status

**Fig 2:**
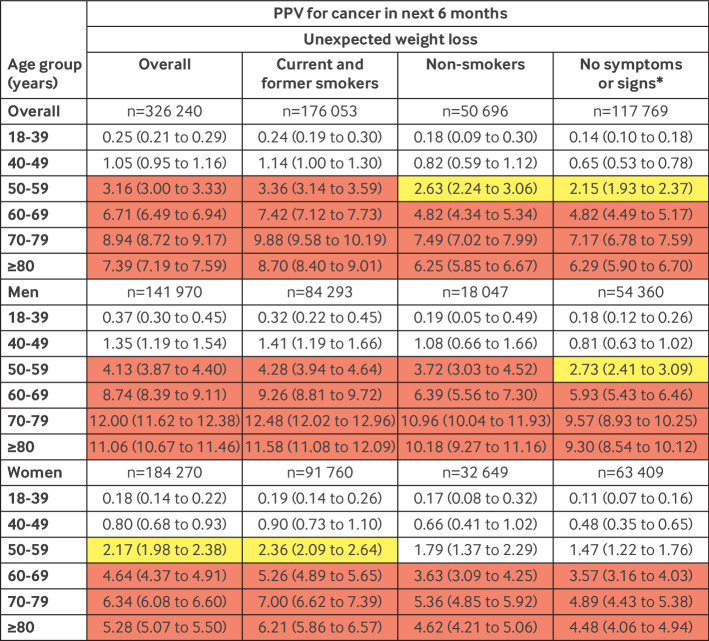
PPVs for cancer by sex, age group, and smoking status. *See table 2 and table 3 for signs and symptoms. Red represents a PPV ≥3%, the threshold above which the National Institute for Health and Care Excellence recommends investigation for cancer. Yellow represents a PPV of 2-3%. PPV=positive predictive value

The PPV for a cancer diagnosis was higher in people who were older and those who smoked (fig 2)—it was >2% in non-smokers aged ≥50 years. Analysis by sex, however, showed that the PPV was >3% for men aged ≥50 years and women aged ≥60 years, regardless of smoking status.

### Signs and symptoms

In multivariable analysis, clinical features selected to be positively associated with cancer in people with unexpected weight loss were abdominal mass, abdominal pain, loss of appetite, back pain, chest signs, dysphagia, fatigue, iron deficiency anaemia, jaundice, lymphadenopathy, rectal mass, and venous thromboembolism (table 2 and table 3). Constipation, abnormal prostate examination, dyspepsia, haemoptysis, hoarseness, and itch were associated with cancer only in men with unexpected weight loss, and nausea, bloating, reflux, and pelvic mass only in women with unexpected weight loss. Positive likelihood ratios in men ranged from 1.43 (95% CI 1.30 to 1.58) for fatigue to 21.00 (8.59 to 51.37) for rectal mass, and in women from 1.28 (1.16 to 1.41) for back pain to 19.46 (12.69 to 29.85) for pelvic mass. Although six symptoms and signs had positive likelihood ratios >5, two of these were relatively uncommon, occurring in 10 to 35 people with a diagnosis of cancer, depending on sex. Negative likelihood ratios ranged from 0.93 to 1.00, with none <0.20 (table 2, table 3, and table 4).

**Table 2:** Likelihood ratios and diagnostic odds ratios for cancer over six months by clinical features in men aged ≥18 years attending primary care with unexpected weight loss

Clinical features	True positive	False positive	False negative	True negative	Positive likelihood ratio (95% CI)	Negative likelihood ratio (95% CI)	Diagnostic odds ratio (95% CI)
**Symptoms**							
Abdominal pain	963	5977	8500	126 530	2.26 (2.11 to 2.41)	0.94 (0.93 to 0.95)	2.40 (2.23 to 2.58)
Loss of appetite	487	3219	8976	129 288	2.12 (1.93 to 2.32)	0.97 (0.97 to 0.98)	2.18 (1.98 to 2.40)
Back pain	716	6945	8747	125 562	1.44 (1.34 to 1.55)	0.98 (0.97 to 0.98)	1.48 (1.37 to 1.60)
Constipation	737	4831	8726	127 676	2.14 (1.98 to 2.30)	0.96 (0.95 to 0.96)	2.23 (2.06 to 2.42)
Dyspepsia	411	2625	9052	129 882	2.19 (1.98 to 2.43)	0.98 (0.97 to 0.98)	2.25 (2.02 to 2.50)
Dysphagia	304	1357	9159	131 150	3.14 (2.77 to 3.55)	0.98 (0.97 to 0.98)	3.21 (2.83 to 3.64)
Fatigue	435	4248	9028	128 259	1.43 (1.30 to 1.58)	0.99 (0.98 to 0.99)	1.45 (1.32 to 1.61)
Haemoptysis	92	534	9371	131 973	2.41 (1.94 to 3.01)	0.99 (0.99 to 1.00)	2.43 (1.94 to 3.03)
Hoarseness	80	406	9383	132 101	2.76 (2.17 to 3.50)	0.99 (0.99 to 1.00)	2.77 (2.18 to 3.53)
Itch	406	1580	9057	130 927	3.60 (3.23 to 4.00)	0.97 (0.96 to 0.97)	3.71 (3.32 to 4.15)
Vomiting	249	1647	9214	130 860	2.12 (1.86 to 2.41)	0.99 (0.98 to 0.99)	2.15 (1.88 to 2.46)
Weakness	82	653	9381	131 854	1.76 (1.40 to 2.21)	1.00 (0.99 to 1.00)	1.77 (1.40 to 2.22)
**Signs**							
Abdominal mass	179	400	9284	132 107	6.27 (5.26 to 7.46)	0.98 (0.98 to 0.99)	6.37 (5.33 to 7.60)
Abnormal prostate finding	42	119	9421	132 388	4.94 (3.48 to 7.02)	1.00 (1.00 to 1.00)	4.96 (3.49 to 7.04)
Chest signs	152	468	9311	132 039	4.55 (3.79 to 5.45)	0.99 (0.98 to 0.99)	4.61 (3.83 to 5.54)
Iron deficiency anaemia	311	1356	9152	131 151	3.21 (2.84 to 3.63)	0.98 (0.97 to 0.98)	3.29 (2.90 to 3.72)
Jaundice	140	296	9323	132 211	6.62 (5.42 to 8.09)	0.99 (0.98 to 0.99)	6.71 (5.48 to 8.21)
Lymphadenopathy	102	423	9361	132 084	3.38 (2.72 to 4.19)	0.99 (0.99 to 0.99)	3.40 (2.74 to 4.23)
Rectal mass	12	8	9451	132 499	21.00 (8.59 to 51.37)	1.00 (1.00 to 1.00)	21.03 (8.83 to 50.06)
Venous thromboembolism	114	553	9349	131 954	2.89 (2.36 to 3.53)	0.99 (0.99 to 0.99)	2.91 (2.38 to 3.56)

CI=confidence interval.

**Table 3:** Likelihood ratios and diagnostic odds ratios for cancer over six months by clinical features in women aged ≥18 years attending primary care with unexpected weight loss

Clinical features	True positive	False positive	False negative	True negative	Positive likelihood ratio (95% CI)	Negative likelihood ratio (95% CI)	Diagnostic odds ratio (95% CI)
**Symptoms**							
Abdominal pain	727	10 046	5434	168 063	2.09 (1.95 to 2.25)	0.93 (0.93 to 0.94)	2.24 (2.07 to 2.42)
Loss of appetite	361	4761	5800	173 348	2.19 (1.98 to 2.43)	0.97 (0.96 to 0.97)	2.27 (2.03 to 2.53)
Back pain	415	9365	5746	168 744	1.28 (1.16 to 1.41)	0.98 (0.98 to 0.99)	1.30 (1.18 to 1.44)
Bloating	55	425	6106	177 684	3.74 (2.83 to 4.95)	0.99 (0.99 to 1.00)	3.77 (2.84 to 4.99)
Dysphagia	174	1806	5987	176 303	2.79 (2.39 to 3.25)	0.98 (0.98 to 0.99)	2.84 (2.42 to 3.32)
Fatigue	315	6763	5846	171 346	1.35 (1.21 to 1.50)	0.99 (0.98 to 0.99)	1.37 (1.22 to 1.53)
Nausea	261	3432	5900	174 677	2.20 (1.94 to 2.49)	0.98 (0.97 to 0.98)	2.25 (1.98 to 2.56)
Reflux	204	3125	5957	174 984	1.89 (1.64 to 2.17)	0.98 (0.98 to 0.99)	1.92 (1.66 to 2.21)
Vomiting	222	2843	5939	175 266	2.26 (1.97 to 2.58)	0.98 (0.97 to 0.98)	2.30 (2.01 to 2.65)
**Signs**							
Abdominal mass	131	397	6030	177 712	9.54 (7.84 to 11.60)	0.98 (0.98 to 0.98)	9.72 (7.97 to 11.86)
Chest signs	70	258	6091	177 851	7.84 (6.03 to 10.20)	0.99 (0.99 to 0.99)	7.92 (6.08 to 10.32)
Iron deficiency anaemia	286	2931	5875	175 178	2.82 (2.51 to 3.18)	0.97 (0.96 to 0.97)	2.91 (2.57 to 3.29)
Jaundice	94	226	6067	177 883	12.02 (9.47 to 15.27)	0.99 (0.98 to 0.99)	12.19 (9.58 to 15.52)
Lymphadenopathy	63	672	6098	177 437	2.71 (2.10 to 3.50)	0.99 (0.99 to 1.00)	2.73 (2.11 to 3.53)
Pelvic mass	35	52	6126	178 057	19.46 (12.69 to 29.85)	0.99 (0.99 to 1.00)	19.56 (12.78 to 29.96)
Rectal mass	10	16	6151	178 093	18.07 (8.20 to 39.80)	1.00 (1.00 to 1.00)	18.10 (8.37 to 39.13)
Venous thromboembolism	94	648	6067	177 461	4.19 (3.38 to 5.20)	0.99 (0.99 to 0.99)	4.24 (3.41 to 5.27)

CI=confidence interval.

**Table 4:** Likelihood ratios and diagnostic odds ratios for cancer over six months by blood tests in men and women aged ≥18 years attending primary care with unexpected weight loss

Symptoms	True positive	False positive	False negative	True negative	Positive likelihood ratio (95% CI)	Negative likelihood ratio (95% CI)	Diagnostic odds ratio (95% CI)
**Liver function tests**							
Low albumin	3001	16 182	7832	172 991	3.24 (3.13 to 3.35)	0.79 (0.78 to 0.80)	4.10 (3.92 to 4.29)
Raised ALP	2994	22 066	7893	176 297	2.47 (2.39 to 2.55)	0.82 (0.81 to 0.83)	3.03 (2.90 to 3.17)
Raised ALT/AST	1425	19 162	9778	177 486	1.31 (1.24 to 1.37)	0.97 (0.96 to 0.97)	1.35 (1.27 to 1.43)
Raised bilirubin	978	18 403	10 322	179 218	0.93 (0.87 to 0.99)	1.01 (1.00 to 1.01)	0.92 (0.86 to 0.99)
**Full blood count**							
Low haemoglobin	5328	35 346	6502	175 909	2.69 (2.63 to 2.75)	0.66 (0.65 to 0.67)	4.08 (3.93 to 4.24)
Raised platelets	2485	13 024	9016	196 927	3.48 (3.35 to 3.62)	0.84 (0.83 to 0.84)	4.17 (3.97 to 4.37)
Raised white cell count	2188	13 108	9455	196 902	3.01 (2.89 to 3.14)	0.87 (0.86 to 0.87)	3.48 (3.31 to 3.65)
**Inflammatory markers**							
Raised CRP	3385	17 141	2102	69 690	3.13 (3.05 to 3.20)	0.48 (0.46 to 0.49)	6.55 (6.18 to 6.93)
Raised ESR	3998	28 802	2336	76 790	2.31 (2.27 to 2.36)	0.51 (0.49 to 0.52)	4.56 (4.33 to 4.81)
**Biochemistry**							
Raised calcium	89	626	6646	103 647	2.20 (1.77 to 2.74)	0.99 (0.99 to 1.00)	2.22 (1.77 to 2.77)
Raised creatinine	3545	50 881	12 079	259 735	1.39 (1.34 to 1.43)	0.92 (0.92 to 0.93)	1.50 (1.44 to 1.56)

ALP=alkaline phosphatase; ALT/AST=alanine transaminase/aspartate transaminase; CI=confidence interval; CRP=C reactive protein; ESR=erythrocyte sedimentation rate.

**Fig 3:**
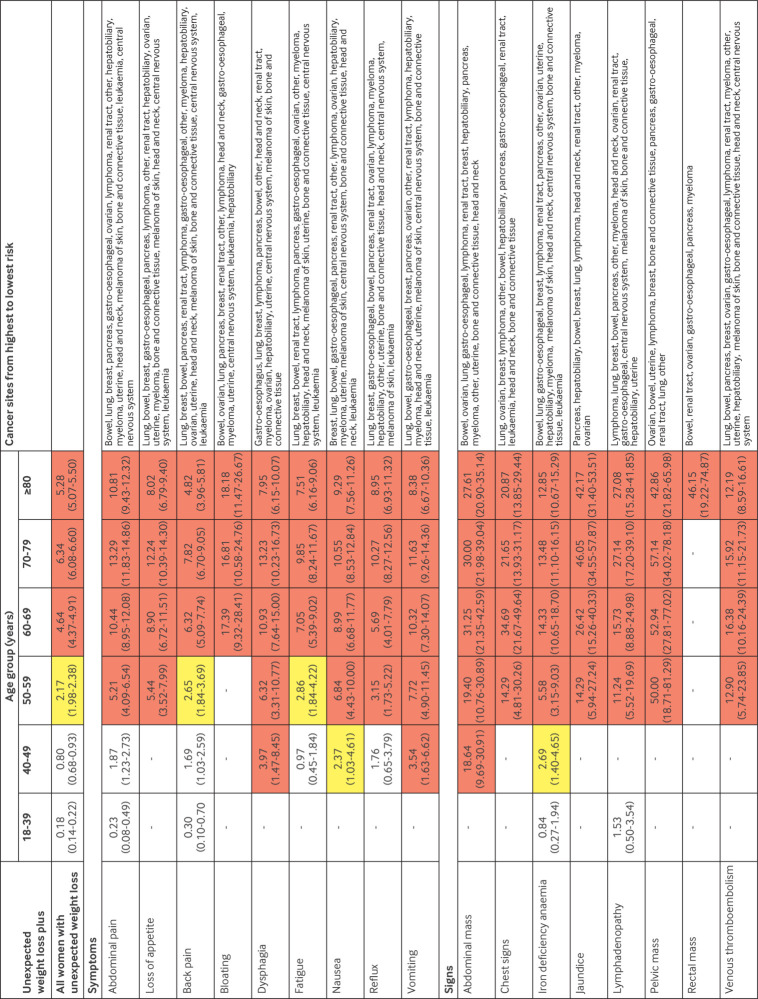
PPVs of symptoms and signs for a cancer diagnosis within six months in women with unexpected weight loss by age group. Red represents a PPV ≥3%, the threshold above which the National Institute for Health and Care Excellence recommends investigation for cancer. Yellow represents a PPV of 2-3%. PPV=positive predictive value

**Fig 4:**
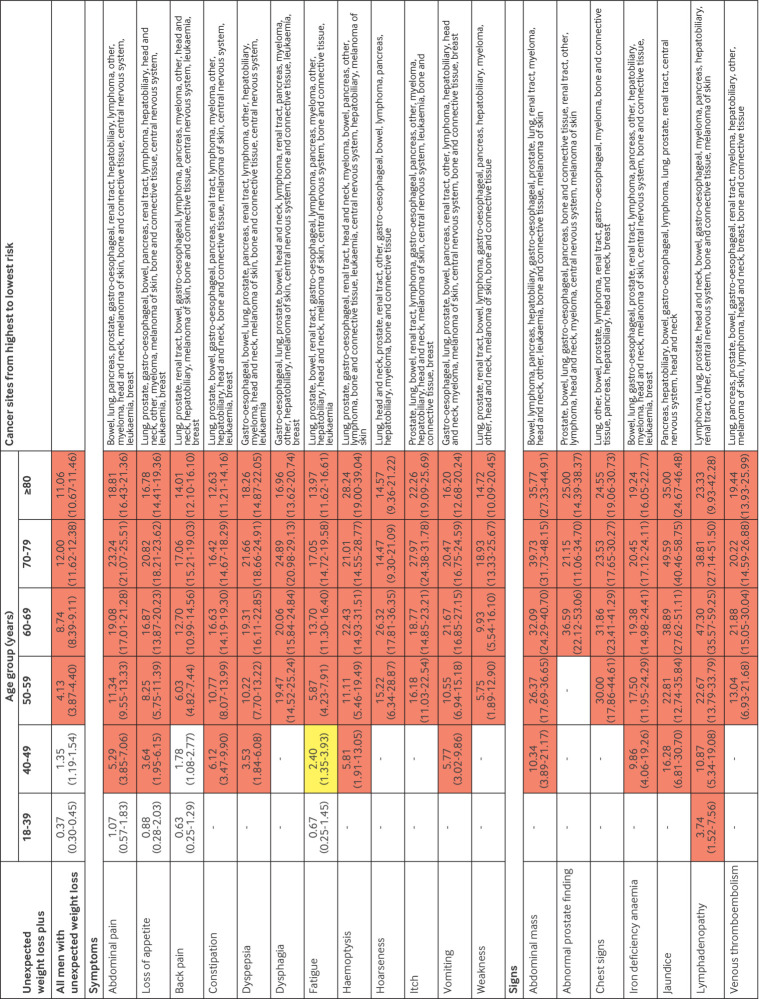
PPVs of symptoms and signs for a cancer diagnosis within six months in men with unexpected weight loss by age group. Red represents a PPV ≥3%, the threshold above which the National Institute of Health and Care Excellence recommends investigation for cancer. Yellow represents a PPV of 2-3%. PPV=positive predictive value

For men and women aged ≥50 years, unexpected weight loss and the co-occurrence of the selected symptoms and signs were associated with a ≥3% increase in the PPV, except for fatigue and back pain in women, which reached this threshold at ages ≥60 years (fig 3 and fig 4). For men aged 40-49 years with unexpected weight loss, the co-occurrence of 10 of the selected symptoms and signs was associated with a ≥3% increase in the PPV, whereas only three reached this level for women in this age group. For men aged 18-39 years with unexpected weight loss, the co-occurrence of lymphadenopathy was also associated with a ≥3% increase in the PPV.

### Blood tests

Several abnormal blood test results in combination with unexpected weight loss had the highest positive likelihood ratio values: low albumin (3.24, 3.13 to 3.35), raised platelets (3.48, 3.35 to 3.62), total white cell count (3.01, 2.89 to 3.14), and raised C reactive protein (3.13, 3.05 to 3.20) (table 4). Normal inflammatory markers had the lowest negative likelihood ratios: C reactive protein (0.48, 95% CI 0.46 to 0.49) and erythrocyte sedimentation rate (0.51, 0.49 to 0.52). Individual blood test results therefore did not reach the ideal threshold of 5 to rule in a cancer diagnosis, or the threshold of 0.2 to rule out a diagnosis.

**Fig 5:**
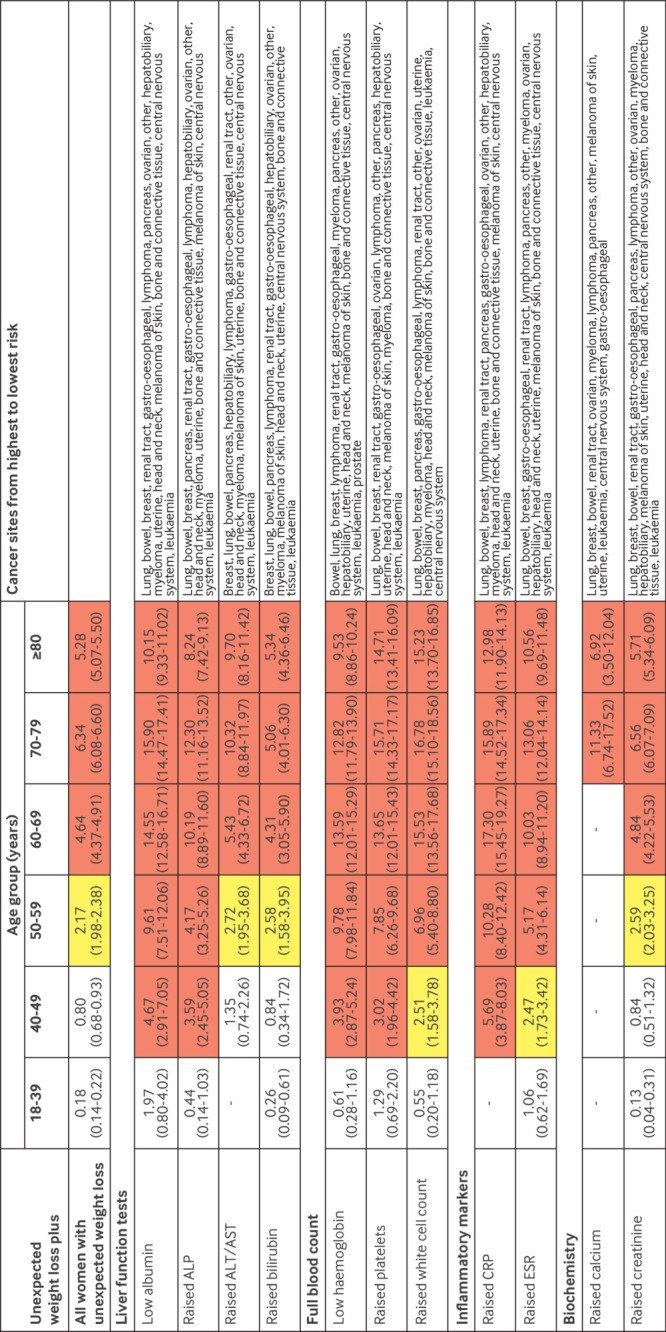
PPVs of blood tests for a cancer diagnosis within six months in women with unexpected weight loss by age group. Red represents a PPV ≥3%, the threshold above which the National Institute for Health and Care Excellence recommends investigation for cancer. Yellow represents a PPV of 2-3%. ALP=alkaline phosphatase; ALT/AST=alanine transaminase/aspartate transaminase; CRP=C reactive protein; ESR=erythrocyte sedimentation rate; PPV=positive predictive value

**Fig 6:**
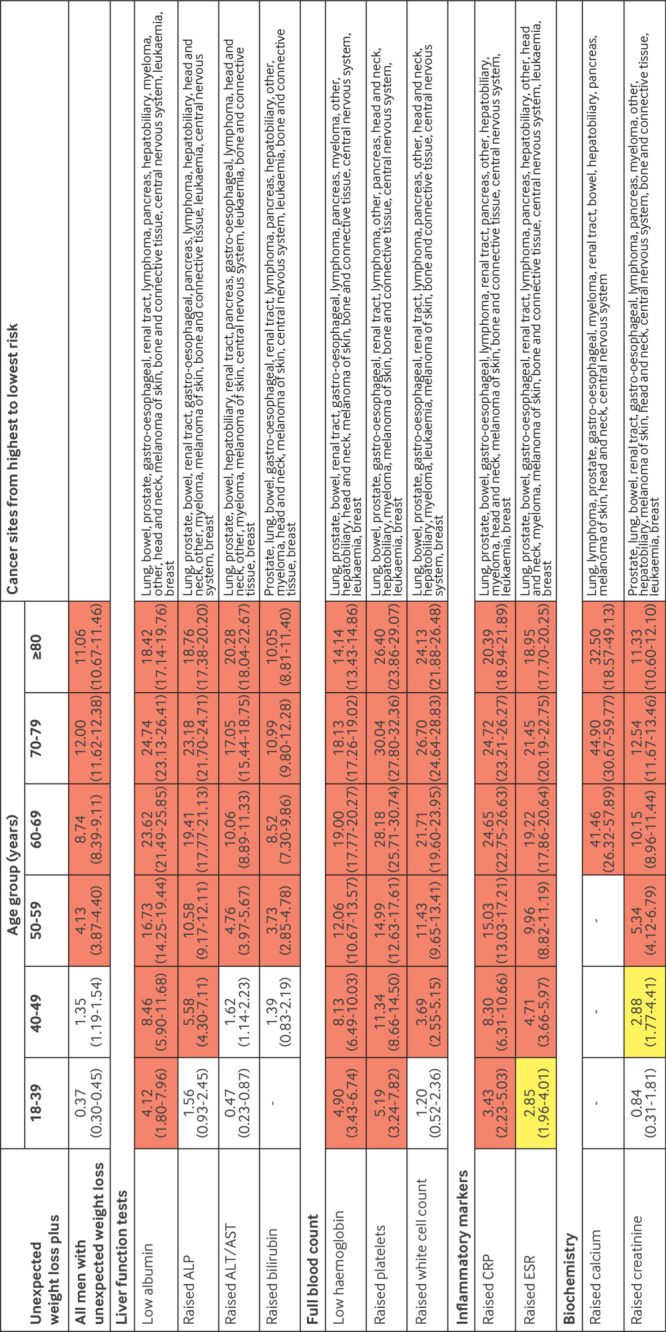
PPVs of blood tests for a cancer diagnosis within six months in men with unexpected weight loss by age group. Red represents a PPV ≥3%, the threshold above which the National Institute of Health and Care Excellence recommends investigation for cancer. Yellow represents a PPV of 2-3%. ALP=alkaline phosphatase; ALT/AST=alanine transaminase/aspartate transaminase; CRP=C reactive protein; ESR=erythrocyte sedimentation rate; PPV=positive predictive value

**Fig 7:**
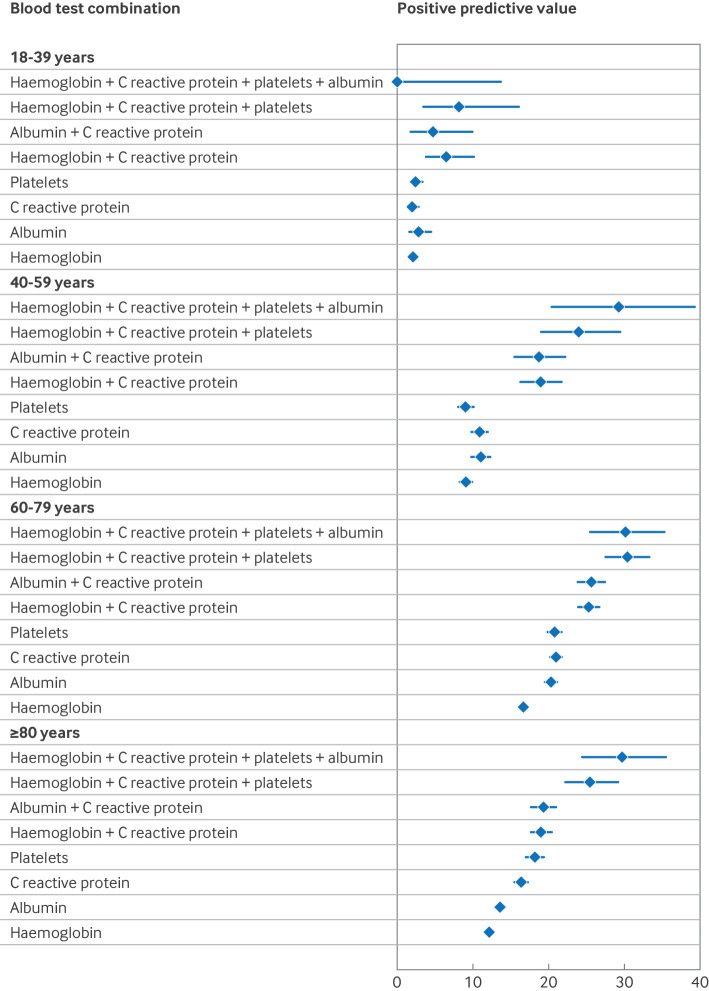
Positive predictive values for combinations of abnormal laboratory test results by age group. Whiskers represent 95% confidence intervals

In men and women aged ≥50 years, however, all individual abnormal blood test results showed a PPV ≥3%, above the underlying PPV for all people with unexpected weight loss (fig 5 and fig 6), except for raised alanine transaminase/aspartate transaminase and creatinine in women (fig 5). For patients aged 40-49 years, PPVs ≥3% were observed for thrombocytosis, low albumin and haemoglobin, and raised alkaline phosphatase and C reactive protein, and in men for raised erythrocyte sedimentation rate and white cell count. Thrombocytosis, low albumin and haemoglobin, and raised C reactive protein in younger men also had PPVs ≥3%. Some combinations of abnormal test results in younger age groups had PPVs ≥3%, but confidence in these estimates was less because not all patients had all tests (fig 7).

### Cancer diagnoses

Cancer was diagnosed in 15 624 (4.8%) patients within six months of the index date, 15 051 (96.3%) of whom were aged ≥50 years and 15 455 (98.9%) ≥40 years. The most frequently diagnosed malignancies were cancers of the lung (n=3569, 22.8%), bowel (n=2434, 15.6%), gastro-oesophagus (n=1936, 12.4%), prostate (n=1373, 8.8%), and pancreas (n=1329, 8.5%). Individually, some clinical features are generally considered to be associated with a single cancer site; however, when they co-occurred with unexpected weight loss, they were associated with several cancer types. For example, men with dyspepsia and unexpected weight loss were diagnosed as having cancers of the following types or site (in rank order): stomach or oesophagus, bowel, lung, prostate, pancreas, renal tract, lymphoma, other, hepatobiliary, myeloma, head and neck, melanoma of skin, bone and connective tissue, central nervous system, and leukaemia (fig 4). Similarly, abnormal test results in patients with unexpected weight loss were also associated with multiple cancer sites. For example, women aged 50-59 years with low albumin levels were diagnosed as having cancers of the following types or site (in rank order): lung, bowel, breast, renal tract, stomach or oesophagus, lymphoma, pancreas, ovarian, other, hepatobiliary, myeloma, uterine, head and neck, melanoma of skin, bone and connective tissue, central nervous system, and leukaemia (fig 5).

### Isolated unexpected weight loss

PPVs for patients without any of the selected clinical features were lower across every age range compared with the full cohort (fig 2). In addition, 81 538 (25.0%) patients had no record of a blood test, 2449 (3.0%) of whom had a diagnosis of cancer. Overall, 867 patients with unexpected weight loss and cancer had neither a clinical feature nor blood test on record: 407 out of 482 men and 334 of 385 women were aged ≥60 years.

### Sensitivity analyses

Appendix 3 shows the results of the sensitivity analysis. Widening the time window made almost no difference to the clinical features selected for inclusion in the final analysis.

### Clinical guideline

Appendix 4 summarises the current NICE guideline recommendations for suspected cancer in patients with unexpected weight loss. Table 5 outlines an updated clinical guideline based on the results of the current analysis.

**Table 5:** Revised clinical recommendations for specialised investigation for cancer in patients with unexpected weight loss

Age (years)	Men and women	Men	Women
18-39	–	Low haemoglobin, lymphadenopathy, hypoalbuminaemia, thrombocytosis, raised C reactive protein	–
40-49	Low haemoglobin, abdominal mass, vomiting, hypoalbuminaemia, raised alkaline phosphatase, raised C reactive protein, thrombocytosis	Abdominal pain, loss of appetite, constipation, dyspepsia, haemoptysis, iron deficiency anaemia, jaundice lymphadenopathy, leucocytosis, raised erythrocyte sedimentation rate	Dysphagia
50-59	–	Anyone with unexpected weight loss	Low haemoglobin, abdominal mass, abdominal pain, loss of appetite, chest signs, dysphagia, hypoalbuminaemia, iron deficiency anaemia, jaundice, leucocytosis, lymphadenopathy, nausea, pelvic mass, raised alkaline phosphatase, raised C reactive protein, raised erythrocyte sedimentation rate, reflux, thrombocytosis, venous thromboembolism, vomiting
≥60	–	Anyone with unexpected weight loss	Anyone with unexpected weight loss

## Discussion

The risk of undiagnosed cancer in adults aged <50 years with recorded unexpected weight loss alone is below the UK’s current 3% threshold that warrants investigation. However, in men aged ≥50 years, women aged ≥60 years, and younger patients with concurrent clinical features (certain symptoms and signs or abnormal results for simple blood tests) the risk of undiagnosed cancer rises above 3%. For women aged 50-59 years these features were low haemoglobin, abdominal mass, abdominal pain, loss of appetite, chest signs, dysphagia, iron deficiency anaemia, jaundice, leucocytosis, lymphadenopathy, low albumin, nausea, pelvic mass, raised alkaline phosphatase, raised C reactive protein, raised erythrocyte sedimentation rate, reflux, thrombocytosis, venous thromboembolism, and vomiting. A subset of 17 clinical features in men and eight in women increased the risk of cancer >3% for patients aged 40-49 years. Low haemoglobin, lymphadenopathy, thrombocytosis, low albumin, and raised C reactive protein in younger men also had PPVs of ≥3%. The absence of individual clinical features in the three months before and one month after the index date, or the presence of individual normal blood test results in this time window, does not reliably rule out cancer in patients with unexpected weight loss.

### Strengths and limitations of this study

We took several steps to improve the likelihood that the unexpected weight loss cohort was accurately defined. Firstly, we confirmed that insufficient weight measurements were recorded in UK primary care to define unexpected weight loss, with clustering of weight recording noted in women with higher BMI and in those with comorbidity.^25^ We then conducted an internal validation study to identify which codes most consistently defined unexpected weight loss, and investigated whether weight measurements could be used in preference to codes.^25^ We included each patient once in the analysis by choosing the first unexpected weight loss code, and excluded patients with a history of cancer to ensure we were investigating unexpected weight loss associated with a first diagnosis of cancer.^6^ We excluded patients with objective evidence of deliberate weight loss (ie, prescription records and coding for bariatric surgery). The presence of advanced disease states might be more likely to be associated with unexpected weight loss and could modify the association of unexpected weight loss and cancer. As it is problematic to identify disease severity using CPRD data, however, we did not exclude patients with these conditions.

We analysed clinical features as occurring with unexpected weight loss if they were coded in the three months before and one month after the index date. This was a clinical decision, as the epidemiology on this topic is limited, based on consideration that a general practitioner is likely to look back at recent notes and investigate unexpected weight loss within a month of presentation. Symptoms occurring more than three months before the index date might well be unlinked to the unexpected weight loss. Some studies have reported the frequency of individual clinical features for people with cancer and controls before a cancer diagnosis, with few symptoms more common in people with cancer than controls earlier than six months before the diagnosis.^16^ ^26^ ^27^ None of these studies, however, reported the timing of multiple symptoms leading up to a cancer diagnosis. We have shown previously that cancers are likely to be diagnosed in patients within three months of presentation with unexpected weight loss, and our findings did not change significantly in sensitivity analysis extending the period to capture co-occurring symptoms and signs up to the day of cancer diagnosis.^1^ The high number of false negatives observed for individual symptoms and signs indicates that many people with cancer do not develop unexpected weight loss and that symptoms and signs are only recorded if patients experience them, patients remember to report them, or they are uncovered by the doctor during a clinical examination.

We also conducted individual analyses for each blood test, including only patients with a result for that blood test. Previous studies have replaced missing blood tests with negative results to allow full case multivariable analysis. We decided against this for two reasons. Firstly, patients who have been tested represent a higher risk population than those who have not been tested,^18^ ^28^ and therefore people with a normal test result might not have the same likelihood of undiagnosed cancer as people who have not been tested. It is unclear how this testing bias relates to the study participants with unexpected weight loss, for whom the blood test was taken close enough to the index date for us to be confident that the test and the result pertained to it. Secondly, classifying absent tests as negative results inflates the number of “true” negatives and misestimates diagnostic accuracy, making it difficult to interpret negative likelihood ratios and negative predictive values. However, as not all patients had been tested with all blood tests, we could not calculate precise estimates for combinations of multiple blood test results. We also dichotomised continuous test results at thresholds used in clinical practice to signify an abnormal result. By dichotomising we lose information by classifying markedly abnormal test results together with mildly abnormal results. The PPVs presented can therefore be considered conservative estimates of the associated cancer risk. The high number of false positive blood test results represent doctors deciding to use blood tests to investigate unexpected weight loss in most patients, that cancer was associated with unexpected weight loss in fewer than 2% of patients with cancer, and that abnormal results in the blood tests studied are not only found in people with cancer. Finally, we classified people as having cancer if a code was entered within six months of presenting with unexpected weight loss. Previous research has shown that if cancer is not diagnosed within six months, the risk of cancer being the cause of the unexpected weight loss is low.^1^ ^29^

### Comparison with other studies

A 2018 systematic review reported higher PPVs of unexpected weight loss than we found here.^4^ This could be accounted for by the considerable heterogeneity between studies included in that review. For example, sensitivity was higher in studies at risk of recall bias. PPVs also varied by the method of data collection, and they were higher in case-control studies than in cohort studies reporting on the same tumour site. A recent clinical review reported 17 symptoms, signs, and test results that in combination with unexpected weight loss had a PPV for cancer of >3%.^2^ These estimates were taken from case-control studies using primary care records that included clinical features occurring in the 1-2 years before the diagnosis of a specific cancer. We studied a much shorter interval around the presentation with unexpected weight loss, included all cancer sites, and had a study size sufficient to allow separate estimates to be produced for each age group and by smoking status and sex. We found evidence for clinical features and abnormal blood test results that were predictive of cancer in combination with unexpected weight loss not previously reported: loss of appetite, abdominal mass, back pain, bloating, chest signs, dyspepsia, fatigue, nausea, reflux, pelvic mass, venous thromboembolism, raised alkaline phosphatase, low albumin, and a raised white cell count. In addition, our study confirmed the importance of abdominal pain, constipation, jaundice, lymphadenopathy, haemoptysis, dysphagia, rectal mass, thrombocytosis, and low haemoglobin, but the implication of these features co-occurring with unexpected weight loss differed from when they occurred alone.

### Policy implications

The risk of cancer in younger adults (<50 years) presenting with unexpected weight loss alone is below the threshold for referral for invasive cancer investigation set by NICE. However, in men aged >50 years, women aged >60 years, and younger patients with the concurrent clinical features shown in fig 3, fig 4, fig 5, and fig 6, the risk of cancer increases such that referral for invasive investigation becomes justified. In the absence of these features, these results might suggest that doctors arrange simple routine blood tests, particularly for full blood count, liver function, erythrocyte sedimentation rate, C reactive protein, and calcium (fig 3 and fig 4). Almost any abnormal test result increases the risk of cancer sufficiently to trigger invasive testing. A higher or lower threshold of cancer risk could be chosen to trigger cancer investigation by primary care clinicians practising outside the UK. The PPVs presented will allow clinicians worldwide to use whichever threshold applies locally. However, as negative likelihood ratios were never <0.20, and although normal blood test results might reassure patients, clinicians should be aware that in isolation a normal blood test result does not reduce the probability of cancer downward enough to rule out the disease in patients with unexpected weight loss.^24^

A pro-inflammatory state underpins cancer cachexia,^30^ ^31^ and prognostic scores composed of markers of the systemic inflammatory response are used in patients with cachexia to predict survival and response to treatment in secondary care.^32^ ^33^ ^34^ A potential avenue for future research is to investigate the utility of inflammatory marker scores and combinations of negative test results in selecting who should (and who should not) undergo invasive testing for cancer.

These findings might also have implications for cancer referral pathways. For example, NICE guidelines suggest that patients with unexpected weight loss and abdominal pain should be investigated for colorectal cancer (see supplementary appendix 4).^2^ In the current study, more than 10 additional cancers presented in this way that would be missed by colonoscopy (fig 3 and fig 4). Likewise, some non-alarm symptoms, such as loss of appetite and fatigue, indicated a probability of cancer that was above the threshold for invasive investigation in the presence of unexpected weight loss. Throughout Denmark and in an increasing number of centres in the UK, Rapid Diagnostic Centres have started to operate that rapidly investigate non-specific symptoms across a broad range of cancer sites^35^ ^36^ ^37^ ^38^ and other associated serious non-cancer conditions.^39^ ^40^

Lastly, women presenting with unexpected weight loss were at markedly lower risk of having cancer than men with unexpected weight loss. Different, but interrelated, mechanisms might underpin this finding. Firstly, women might be more likely to visit their doctor to discuss their weight: they are also more likely to have a weight measurement recorded in UK primary care.^25^ Secondly, women may be more likely to report symptoms of cancer earlier to prompt investigation before weight loss occurs. Thirdly, men may delay presentation until weight loss is noticeable.^41^ This study could not examine these possibilities. Nevertheless, routine weight measurement in primary care could lead to the earlier detection of weight change.

### Conclusion

Unexpected weight loss alone in people aged <50 years is unlikely to be due to cancer, and immediate referral for invasive testing might not be justified. In men aged ≥50 years and women aged ≥60 years, onward referral might be justified without additional clinical features recorded in the three months before and one month after the index date. Some additional clinical features recorded for younger patients in this time window increase the risk of cancer substantially over the 3% threshold, justifying further investigation. Clinical features thought to be specific to an individual cancer site are markers of several different types of cancer when they co-occur with unexpected weight loss, which could mean that new, broader investigative approaches are needed for patients with unexpected weight loss.

**What is already known on this topic**

The likelihood of a diagnosis of early or late stage cancer is increased in the 3-6 months after the first record of unexpected weight loss in primary care
Malignancies most strongly predicted by unexpected weight loss are lymphoma, cancer of unknown primary, or pancreatic, gastro-oesophageal, hepatobiliary, lung, bowel, and renal tract cancers
Studies that have investigated the predictive value of clinical features in combination with unexpected weight loss have not acknowledged that predictive values vary during different periods

**What this study adds**

The risk of undiagnosed cancer in young adults (<50 years) attending primary care with unexpected weight loss alone is below the UK’s current 3% threshold warranting investigation
In men aged ≥50 years, women aged ≥60 years, and younger patients with other clinical features that could indicate cancer, the risk of undiagnosed cancer across multiple sites rises above the 3% threshold
For women aged 50-59 years these features were low haemoglobin, abdominal mass, abdominal pain, loss of appetite, chest signs, dysphagia, iron deficiency anaemia, jaundice, leucocytosis, lymphadenopathy, low albumin, nausea, pelvic mass, raised alkaline phosphatase, raised C reactive protein, raised erythrocyte sedimentation rate, reflux, thrombocytosis, venous thromboembolism, and vomiting

## Data Availability

This study is based on CPRD data and is subject to a full licence agreement, which does not permit data sharing outside of the research team. Code lists are available from the corresponding author.

## Web extra

Extra material supplied by authors

Supplementary information: appendices 1-4

## Notes

Contributors: BDN (principal investigator) conceived and oversaw the study, wrote the protocol, developed the code lists for use in the study, conducted data management, conducted statistical analysis with guidance from CK and PV, and interpreted the statistical analysis. PV conducted data management and statistical analysis. SJP collated the CPRD code lists for the features of cancer. BDN wrote the first draft of the manuscript. All members of the team were involved in the drafting and commenting on further revisions of the manuscript. All authors read and approved the final manuscript. BDN is the guarantor. The corresponding author attests that all listed authors meet authorship criteria and that no others meeting the criteria have been omitted.

Transparency: The lead author (BDN) affirms that this manuscript is an honest, accurate, and transparent account of the study being reported; that no important aspects of the study have been omitted; and that any discrepancies from the study as planned (and, if relevant, registered) have been explained.

Dissemination to participants and related patient and public communities: The results of this study will be disseminated through the media channels of the host institution of the lead author and the funder for scientific and lay audiences.

Provenance and peer review: Not commissioned; externally peer reviewed.
