# Supplementary material for: Prioritising primary care patients with unexpected weight loss for cancer investigation: diagnostic accuracy study (update)
Source: BMJ. 2024 Oct 16;387:e080199. doi: 10.1136/bmj-2024-080199 (PMC11480917; doi:10.1136/bmj-2024-080199)
Supplement: Supplementary file 1 — Supplementary information: appendices 1-4 [file nicb080199.ww.pdf]

Supplementary Material

Prioritising primary care patients with unexpected weight loss for cancer investigation: diagnostic accuracy study (update).

Nicholson BD et al.

Appendix 1: **Backwards stepwise selection of covariates associated with cancer diagnosis within six months in adults attending primary care with UWL.**

**Symptom window**	**Minus three plus one month of the UWL code**		**Minus three plus three months of the UWL code**	
Sex	**Males**	**Females**	**Males**	**Females**
Age (ref=60-69)				
18-39	0.04 (0.03, 0.05)	0.04 (0.03, 0.05)	0.04 (0.03, 0.05)	0.04 (0.03, 0.05)
40-49	0.14 (0.11, 0.16)	0.16 (0.13, 0.20)	0.14 (0.12, 0.16)	0.16 (0.13, 0.20)
50-59	0.44 (0.40, 0.49)	0.44 (0.39, 0.50)	0.44 (0.40, 0.49)	0.44 (0.38, 0.50)
70-79	1.51 (1.41, 1.62)	1.41 (1.29, 1.54)	1.50 (1.40, 1.60)	1.40 (1.28, 1.54)
80+	1.45 (1.35, 1.56)	1.36 (1.24, 1.49)	1.43 (1.33, 1.54)	1.35 (1.23, 1.48)
BMI (ref=normal)				
Underweight	0.94 (0.82, 1.08)	0.92 (0.81, 1.04)	0.94 (0.82, 1.07)	0.92 (0.81, 1.04)
Overweight	1.21 (1.14, 1.28)	1.25 (1.16, 1.35)	1.21 (1.14, 1.28)	1.24 (1.16, 1.34)
Obese	1.15 (1.07, 1.24)	1.16 (1.06, 1.27)	1.15 (1.06, 1.24)	1.16 (1.06, 1.27)
Smoking (ref=non)				
Current	1.43 (1.33, 1.54)	1.57 (1.45, 1.70)	1.44 (1.33, 1.55)	1.58 (1.45, 1.71)
Past	1.10 (1.02, 1.19)	1.20 (1.10, 1.30)	1.10 (1.03, 1.19)	1.20 (1.11, 1.31)
Symptom (ref=no)				
Appetite Loss	1.45 (1.28, 1.64)	1.31 (1.13, 1.52)	1.42 (1.26, 1.61)	1.29 (1.12, 1.50)
Pain Shoulder		0.73 (0.58, 0.92)		0.70 (0.56, 0.89)
Oral Symptoms	0.65 (0.49, 0.87)			
Constipation	1.33 (1.20, 1.46)		1.58 (1.45, 1.71)	1.34 (1.19, 1.50)
Pain Chest				
Chest Signs	2.51 (2.00, 3.15)	4.36 (3.13, 6.07)	2.50 (1.99, 3.13)	4.34 (3.11, 6.05)
Seizure	0.44 (0.26, 0.75)			
Weakness	1.44 (1.10, 1.88)		1.56 (1.25, 1.96)	
Diarrhoea				
Dyspepsia	1.67 (1.46, 1.91)		1.64 (1.44, 1.88)	
Dysphagia	2.30 (1.98, 2.68)	1.98 (1.63, 2.41)	2.25 (1.93, 2.62)	1.98 (1.63, 2.40)
Dysuria		0.50 (0.32, 0.77)		0.60 (0.42, 0.85)
Nausea		1.52 (1.29, 1.79)		1.50 (1.27, 1.77)

Distension/bloating		1.94 (1.32, 2.85)		2.05 (1.44, 2.92)
Reflux		1.38 (1.15, 1.65)		1.36 (1.14, 1.63)
Abnormal Prostate Examination	1.85 (1.16, 2.93)			
Pain abdomen	2.09 (1.91, 2.28)	1.96 (1.77, 2.18)	2.05 (1.87, 2.24)	1.92 (1.73, 2.13)
Pain back	1.38 (1.25, 1.51)	1.18 (1.04, 1.33)	1.35 (1.23, 1.49)	
Fatigue	1.29 (1.14, 1.46)	1.24 (1.07, 1.44)	1.27 (1.12, 1.44)	1.23 (1.05, 1.43)
Venous Thromboembolism	2.14 (1.68, 2.72)	2.61 (1.99, 3.43)	2.14 (1.68, 2.73)	2.61 (1.99, 3.43)
Memory Disturbance	0.59 (0.46, 0.74)		0.57 (0.46, 0.70)	0.72 (0.57, 0.90)
Headache		0.57 (0.41, 0.81)		0.58 (0.41, 0.81)
Mass Pelvis		10.86 (5.89, 20.04)		11.49 (7.20, 18.34)
Haemoptysis	2.10 (1.59, 2.78)		2.04 (1.54, 2.71)	
Hoarse Voice	1.57 (1.17, 2.11)		1.56 (1.19, 2.04)	
Night sweats				
Mass Abdomen	3.77 (2.99, 4.76)	5.00 (3.74, 6.68)	3.81 (3.02, 4.80)	4.89 (3.66, 6.54)
Iron Deficiency Anaemia	2.03 (1.75, 2.36)	2.32 (1.96, 2.74)	2.01 (1.73, 2.34)	2.31 (1.96, 2.73)
Visual disturbance			0.65 (0.47, 0.89)	
Vomiting	1.58 (1.33, 1.88)	1.49 (1.24, 1.79)	1.57 (1.32, 1.86)	1.47 (1.22, 1.76)
Jaundice	5.14 (3.93, 6.72)	7.35 (5.31, 10.17)	5.08 (3.88, 6.65)	7.07 (5.11, 9.79)
Mass Rectum	10.43 (3.28, 33.12)	4.83 (1.50, 15.59)	10.68 (3.67, 31.06)	4.73 (1.46, 15.31)
Pruritis Itch	2.58 (2.26, 2.96)		2.41 (2.14, 2.72)	
Shortness Of Breath	0.82 (0.75, 0.89)		0.81 (0.74, 0.89)	
Lymphadenopathy	4.86 (3.62, 6.52)	3.71 (2.61, 5.27)	4.72 (3.51, 6.34)	3.56 (2.50, 5.06)

Full female models before stepwise selection. Outcome: *cancer diagnosis within six months.* Categorical covariates: *age-group, smoking status, BMI group, alcohol consumption;* Binary covariates: *abdominal pain, appetite loss, bleeding gums, breast symptom- lump, breast symptom – other, breast symptom – skin, change in bowel habit, constipation, cough, chest signs, diarrhoea, distension, dyspepsia, dysphagia, dysuria, dizzy, digital rectal examination, epistaxis, fatigue, fever, frequency, haematemesis haematuria, haemoptysis, hoarse voice, headache, iron deficiency anaemia, infection – chest, infection – recurrent, jaundice, limp, lump, lower urinary tract symptoms, lymphadenopathy, memory problems, mass - abdomen, mass - pelvis, mass - rectum, mass – vagina, mass - vulva, nausea, night sweats, non-cardiac chest pain, non-visible haematuria, nipple discharge, nipple retraction, oral symptoms, pain after alcohol, pain – back, pain – breast, pain – bone, pain – pelvis, pain – shoulder tip, pain – throat, proteinuria, pruritus, rectal bleeding, reflux , skin lesion, speech problems , seizure, shortness of breath, steatorrhoea, stridor , urgency of micturition , urinary tract infection, ulcer – vulva, vaginal discharge, venous thromboembolism, visual disturbance, vomiting, weakness.*
Full male models before stepwise selection. *Outcome: cancer diagnosis within six months.* Categorical covariates*: age-group, smoking status, BMI group, alcohol consumption;* Binary covariates*: abdominal pain, abnormal prostate examination, appetite loss, bleeding gums, change in bowel habit, constipation, cough, chest signs, diarrhoea, distension, dyspepsia, dysphagia, dysuria, dizzy, erectile dysfunction, epistaxis, fatigue, fever, frequency, haematemesis haematuria, haemoptysis, hoarse voice, headache, iron deficiency anaemia, infection – chest, infection – recurrent, jaundice, limp, lump, lower urinary tract symptoms, lymphadenopathy, memory problems, mass - abdomen, mass - pelvis, mass - rectum, mass – testis, nausea, night sweats, non-cardiac chest pain, non-visible haematuria, oral symptoms, pain after alcohol, pain – back, pain – bone, pain – pelvis, pain – shoulder tip, pain – throat, proteinuria, pruritus, rectal bleeding, reflux , skin lesion, speech problems , seizure, shortness of breath, steatorrhoea, stridor, testis – change, testis – symptom testis urgency of micturition, urinary tract infection, venous thromboembolism, visual disturbance, vomiting, weakness.*

Appendix 2**. The ten most commonly reported laboratory tests in the three months prior to one month after UWL.**

	Normal range	n/63973 (%) tested	Abnormal	n/N (%) abnormal
Liver function tests				
**Albumin**	35-50 g/L	41622 (65.06)	Low	2867/41622 (6.9)
**Alkaline Phosphatase**	30-130 iu/L	41278 (64.52)	Raised	7024/41278 (17)
**Bilirubin**	3-17 umol/L	41895 (65.49)	Raised	2075/41895 (5)
Full Blood Count				
**Haemoglobin**	130-180 g/L (male)<br>115-160 g/L (female)	46129 (72. 1)	Low	7440/46129 (16.1)
**Total white cell count**	4.0-11.00 x10^9/L	44714 (69.9)	Raised	3245/44714 (13.8)
**Platelets**	150-400 x10^9/L	45239 (70.7)	Raised	1903/45239 (4.2)
Inflammatory markers				
**C-reactive protein**	0-10 mg/L	14703 (23.0)	Raised	3085/14703 (21.0)
**Erythrocyte sedimentation rate**	0-10 mm/h (aged <65 years, male)<br>0-15 mm/h (aged <65 years, female)<br>0-20 mm/h (aged ≥65 years, male)<br>0-25 mm/h (aged ≥65 years, female)	22931 (35.9)	Raised	6785/22931 (33.6)
Biochemistry				
**Calcium**	2.12-2.60 mmol/L	20214 (31.6)	Raised	429/20214 (2.12)
**Creatinine**	60-106 umol/L (male)<br>45- 80 umol/L (female)	44671 (69.8)	Raised	13225/44671 (29.6)

Appendix 3. **Clinical features with a prevalence ≥1% in the three months prior to one month following UWL.**

		**Weight loss**
		n / 326,240 (%)
**Clinical features**	Cough	21891 (6.7)
	Abdominal Pain	17713 (5.4)
	Back Pain	17441 (5.3)
	Shortness Of Breath	17239 (5.3)
	Infection Chest	15344 (4.7)
	Constipation	12212 (3.7)
	Fatigue	11761 (3.6)
	Diarrhoea	11196 (3.4)
	Urinary Tract Infection	9494 (2.9)
	Postmenopausal Bleeding	9200 (2.8)
	Appetite Loss	8828 (2.7)
	Chest Pain	8111 (2.5)
	Dyspepsia	6813 (2.1)
	Lower Urinary Tract Symptoms	6632 (2.0)
	Shoulder Pain	6367 (2.0)
	Dizziness	6266 (1.9)
	Reflux	6151 (1.9)
	Non-Visible Haematuria	5644 (1.7)
	Nausea	5187 (1.6)
	Vomiting	4961 (1.5)
	Iron Deficiency Anaemia	4884 (1.5)
	Headache	4329 (1.3)
	Sore Throat	3994 (1.2)
	Haematuria	3811 (1.2)
	Change In Bowel Habit	3804 (1.2)
	Memory Disturbance	3796 (1.2)
	Itch	3741 (1.1)
	Dysphagia	3641 (1.1)
	Bleeding Rectal	3314 (1.0)
	Oral Symptoms	3252 (1.0)

Appendix 4. **Current NICE recommendations for the investigation of cancer in patients with UWL (3, 10).**

**Age**	**Unexpected weight loss AND:**	**Action**	**Cancer**
**N/A**	lymphadenopathy or splenomegaly	Urgent specialist referral*	Lymphoma
**N/A**	female	CA125 test in primary care	Ovarian
**≥40y**	abdominal pain	Urgent specialist referral*	Colorectal
	ever-smoker / asbestos exposure	Urgent Chest X-Ray	Lung
	never-smoker AND cough / fatigue / shortness of breath / chest pain / appetite loss		
**<50y**	rectal bleeding	Consider urgent specialist referral	Colorectal
**≥50y**		Faecal Occult Blood Test	
**≥55y**	upper abdominal pain / reflux / dyspepsia	Urgent Gastroscopy	Gastro -oesophageal
	thrombocytosis / nausea and vomiting	Non-urgent Gastroscopy	
**≥60y**	Diarrhoea / back pain / abdominal pain / nausea / vomiting / constipation / new onset diabetes.	Consider urgent CT Abdomen	Pancreas

**to be assessed within 2 weeks*
